# Synthesis of Shape-Tailored WO_3_ Micro-/Nanocrystals and the Photocatalytic Activity of WO_3_/TiO_2_ Composites

**DOI:** 10.3390/ma9040258

**Published:** 2016-03-31

**Authors:** István Székely, Gábor Kovács, Lucian Baia, Virginia Danciu, Zsolt Pap

**Affiliations:** 1 Faculty of Chemistry and Chemical Engineering, Babeș-Bolyai University, Arany János 11, Cluj-Napoca RO-400028, Romania; szistike@yahoo.com (I.S.); vdanciu@chem.ubbcluj.ro (V.D.); 2 Faculty of Physics, Babeș–Bolyai University, M. Kogălniceanu 1, Cluj–Napoca RO-400084, Romania; gkovacs@chem.ubbcluj.ro (G.K.); lucian.baia@phys.ubbcluj.ro (L.B.); 3 Department of Applied and Environmental Chemistry, University of Szeged, Rerrich Béla tér 1, Szeged HU-6720, Hungary; 4 Institute for Interdisciplinary Research on Bio-Nano-Sciences, Treboniu Laurian 42, Cluj-Napoca RO-400271, Romania; 5 Institute of Environmental Science and Technology, Tisza Lajos krt. 103, Szeged HU-6720, Hungary

**Keywords:** hydrothermal crystallization, WO_3_ nanocrystallites, WO_3_/TiO_2_ nanocomposites, photocatalytic activity, shape tuning/tailoring

## Abstract

A traditional semiconductor (WO_3_) was synthesized from different precursors via hydrothermal crystallization targeting the achievement of three different crystal shapes (nanoplates, nanorods and nanostars). The obtained WO_3_ microcrystals were analyzed by the means of X-ray diffraction (XRD), scanning electron microscopy (SEM) and diffuse reflectance spectroscopy (DRS). These methods contributed to the detailed analysis of the crystal morphology and structural features. The synthesized bare WO_3_ photocatalysts were totally inactive, while the P25/WO_3_ composites were efficient under UV light radiation. Furthermore, the maximum achieved activity was even higher than the bare P25’s photocatalytic performance. A correlation was established between the shape of the WO_3_ crystallites and the observed photocatalytic activity registered during the degradation of different substrates by using P25/WO_3_ composites.

## 1. Introduction

WO_3_ is a well-known semiconductor with a large applicability spectrum. Its color can vary from yellow, green, bluish and grayish depending on the oxidation state of the tungsten atoms in the crystal structure. It is a widely studied transition metal oxide with a light absorption maximum ≈ 480 nm (the band gap of WO_3_ is ≈2.6 eV [1], yellowish color), stable under acidic and oxidative conditions and most importantly, it is considered harmless. Over the years, WO_3_ nanomaterials were applied as pigments for paints [2], gas-, humidity- and moisture sensors [3], important components of energy efficient (smart) windows, antiglare automobile rear-view mirrors and sunroofs [4]. WO_3_ is capable of electrochromism, which is an optical modulation between blue color and transparent, a feature that occurs upon ion-electron double injection and extraction [5].

WO_3_ nanocrystallites can be synthesized using various methods, the most common being the ones using hydrothermal crystallization. Tungsten trioxide shows four well-known crystal phases: tetragonal, orthorhombic, monoclinic and triclinic. The most frequently obtained crystal phase is monoclinic [6]. Tungsten trioxide has been widely studied as a potential photocatalyst, although the photoactivity of WO_3_ is relatively low (compared to TiO_2_) [7] and can be significantly enhanced if it is applied in composite systems with noble metals or other semiconductor oxides [8].

The main advantages of WO_3_ are that it can be synthesized relatively easy; it absorbs and reflects light (its color can vary from yellow, green to blue and white/grey) at a much broader spectral range compared to TiO_2_. The band-gap of WO_3_ is narrower compared to TiO_2_, which means that WO_3_ requires lower energy photons in the heterogeneous photocatalytic process [9].

The synthesis of WO_3_ semiconductors has received significant attention in the last few years. Most of these studies were focused on the morphology of WO_3_ obtained by hydrothermal crystallization, which is a frequently used preparation procedure. In some cases, it can behave also as a charge separator [10] meaning that this semiconductor can enhance other semiconductors’ charge separation efficiency; therefore, it is a viable option for composite systems [11,12,13]. WO_3_ can form composites with noble metals such as Pt, Au or with other semiconductors like TiO_2_, ZnO or even NiO. The most widely used combinations are those with TiO_2_ and noble metals. The above listed composites were used as gas sensors [14,15] or as very efficient photocatalysts [16].

The morphology of WO_3_ can be influenced with the temperature of the hydrothermal crystallization, the precursors’ structure and solvent’s polarity, pH, *etc.* [17,18,19]. Tungsten trioxide can be synthesized starting from a larger variety of precursors including: tungstic acid, sodium tungstate and ammonium metatungstate. These compounds were already proved to yield different crystal geometries of WO_3_ [1,20,21].

In this work, WO_3_ photocatalysts were obtained from three different precursors via hydrothermal crystallization. The morphology, structure and photocatalytic activity of WO_3_ were studied and WO_3_/TiO_2_ composites were prepared and their photoactivity was evaluated and the activity-morphology-structure relationship was established.

## 2. Results

### 2.1. Photocatalytic Activity

**Figure 1:**
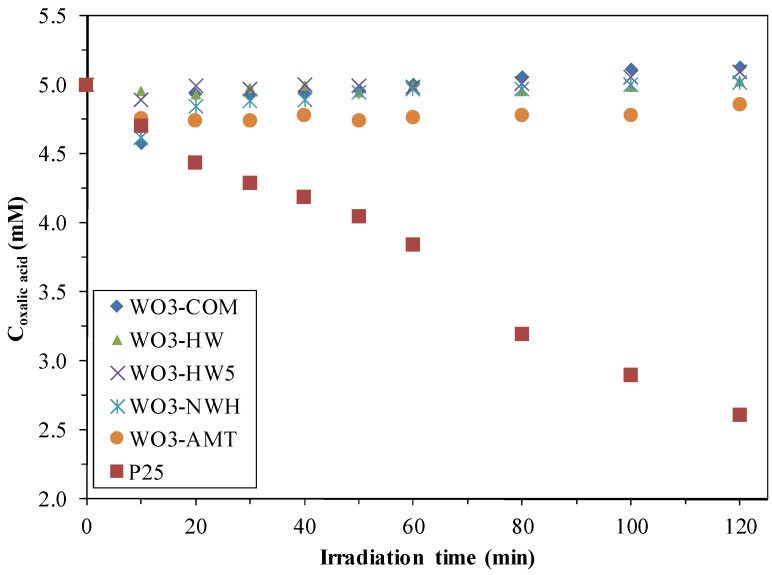
Photodegradation of oxalic acid under UV light using bare WO_3_.

**Figure 2:**
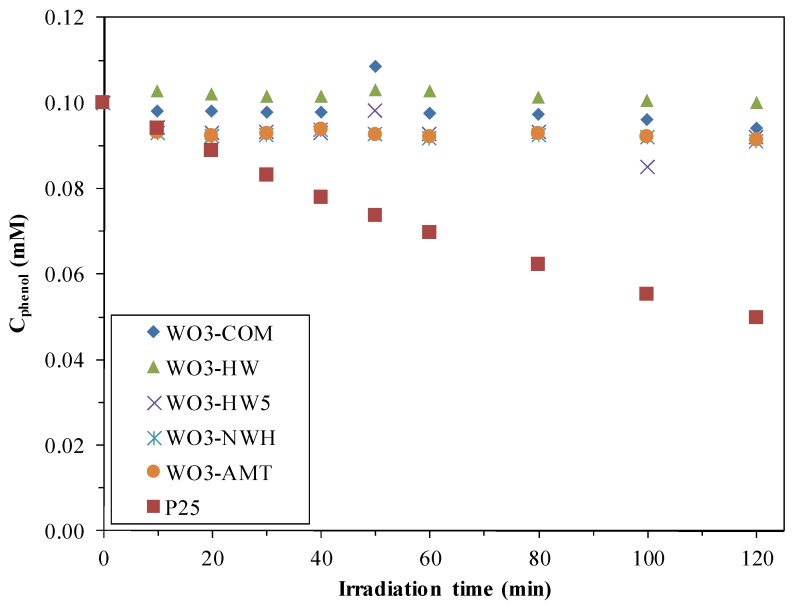
Photode gradation of phenol under visible light with WO_3_ microcrystallites.

Data from the literature shows that WO_3_ photocatalysts’ activity was usually very low, excepting some specific cases [18]. To verify this, the photocatalytic activity of the bare WO_3_ nanocrystals were tested both under UV and visible light. As Figure 1 and Figure 2 shows, (only the degradation of phenol under visible light and of oxalic acid under UV light are shown) the synthesized semiconductors were not active compared to Evonik Aeroxide P25 TiO_2_ (later on, the commercial product will be denoted as P25), which was active also under both visible- and UV light. The visible light activity can be attributed to the presence of a small fraction of rutile crystal phase in P25 [22,23].

The inactivity of the WO_3_ nano- and microcrystals possibly resides in the following issues:

a.) large particle size of the synthesized WO_3_. Although the obtained microcrystals have hierarchical structure, their secondary morphology was in the micrometer range. It is already known in the case of titania that, over a certain particle size, the overall photocatalytic activity decreases (some of the largest titania crystals which are known to have good photocatalytic activity are Aldrich rutile and Aldrich anatase, each of them having a crystal size above 100 nm [24].

b.) the absence of an electron acceptor. In some cases, an electron acceptor (e.g., noble metal nanocrystals) can enhance the activity of a semiconductor [6], which was missing from our composite system (from the WO_3_’s point of view).

As it can be seen, the bare WO_3_ crystallites are not active at all under UV/visible light. However, WO_3_ is known also for electrochromic properties, which are based on its electron acceptor capacity. This was exploited in composite systems in which TiO_2_ is in contact with WO_3_. The composites were obtained according to the Section 4.3.

The best way to examine the influence of the chosen WO_3_s on the photocatalytic activity of titania is to choose a very active, vastly documented photocatalyst. Therefore, the best option is P25. It is known to degrade the majority of organic contaminants (phenol, 4-chlorophenol, dichloroacetic acid, dimethylamine, trichloroethylene, acid orange 7, methylene blue, methanol, *etc.* [25]) and is considered the most unselective photocatalyst, producing lower amounts of intermediate compounds.

### 2.2. Phenol Conversion Rates

**Figure 3:**
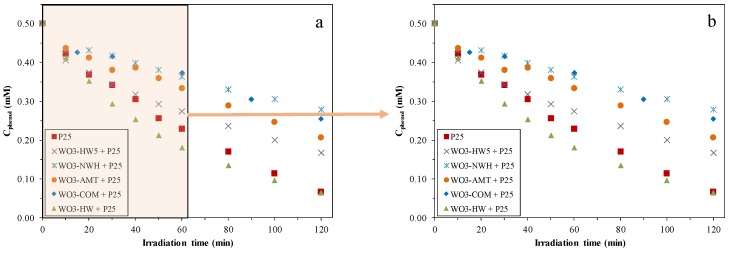
Degradation curves of phenol using WO_3_-P25 composites under UV light, after 2 h (**a**); and 1 h (**b**).

After 1 h, P25 degraded 54.3% of the total phenol concentration. From Figure 3, it can be observed that there were two types of composites (the short names for the obtained WO_3_s can be found in the experimental section). Some of their efficiency was lower than the efficiency of P25: WO_3_-NWH + P25 (27.5% degraded phenol), WO_3_-COM + P25 (25.4% degraded phenol), WO_3_-AMT + P25 (33.4% degraded phenol) and WO_3_-HW5 + P25 (45.3% degraded phenol). WO_3_-HW + P25 was the only composite with slightly superior activity compared to P25, showing a 63.9% phenol decomposition efficiency. According to other published data, this result is interesting, as the WO_3_-TiO_2_ composites’ efficiency towards phenol was reported to be lower [6] compared to that of the bare TiO_2_. Higher activity was achieved only when a third composite component (noble metals—Au or Pt) was also introduced or the TiO_2_-WO_3_ interparticle contact was maximized by the adjustment of the semiconductors’ surface charge [16,26,27]. The main reason for which the degradation curves were plotted separately after 1 h and 2 h was that, after one hour, the degradation rates were not influenced significantly by the intermediates’ concentration.

After 2 h, the reference photocatalyst degraded 86.8% of the organic pollutant. WO_3_-NWH + P25 (44.4% degraded phenol), WO_3_-COM + P25 (49.1% degraded phenol), WO_3_-AMT + P25 (58.7% degraded phenol), and WO_3_-HW5 + P25 composite (66.7% degraded phenol) remained less photoactive than P25. WO_3_-HW + P25 was the only composite that showed a comparable efficiency towards phenol degradation, achieving 87.2% degradation. The degradation efficiency values of P25 and WO_3_-HW + P25 were much closer after 2 h (1 h of degradation: 54.3% *vs.* 63.9%; 2 h of degradation: 86.8% *vs.* 87.2%).

### 2.3. Reaction Rates of the Phenol Degradation

The reference photocatalyst showed 8.90 × 10^−3^ mmol·dm^−3^·min^−1^ initial reaction rate, which was inferior compared to WO_3_-COM + P25 – 11.18 × 10^−3^ mmol·dm^−3^·min^−1^. Interestingly, the initial reaction rate of WO_3_-HW + P25 composite was nearly identical with the value shown by P25 – 8.86 × 10^−3^ mmol·dm^−3^·min^−1^. Although the WO_3_-COM + P25 showed the highest initial reaction rate, after 2 h it degraded only 50% of the phenol, while WO_3_-HW + P25 removed 87.2%. The reaction rates of the other composites were noticeably lower than the value obtained for P25. The differences and inconsistencies shown between the degradation yields and initial reaction rates raised the following important aspect: the activity values of the composites were dependent from the chosen model pollutant—representative examples are methylene blue, rhodamine B, malachite green, 2-chloro-phenol, 2-nitro-phenol and phenol, which show an affinity at different levels towards WO_3_ [28,29,30,31,32,33].

Nevertheless, different substrates also mean different degradation pathways. It is remarkable that a pollutant with a relatively simple structure such as phenol itself degrades through different intermediates in different proportions when the same type of composite is applied (the difference in these cases is usually just the composite build-up). However, fortunately, there are common intermediates in the degradation pathways, such as hydrochinon, pyrocatechol and resorcinol [34,35]. The end products, of course, in each of these reactions are water and CO_2_. Hence, in order to get more information about the activity of these nanomaterials, another model pollutant is needed.

### 2.4. Reaction of the Methyl-Orange Degradation

**Figure 4:**
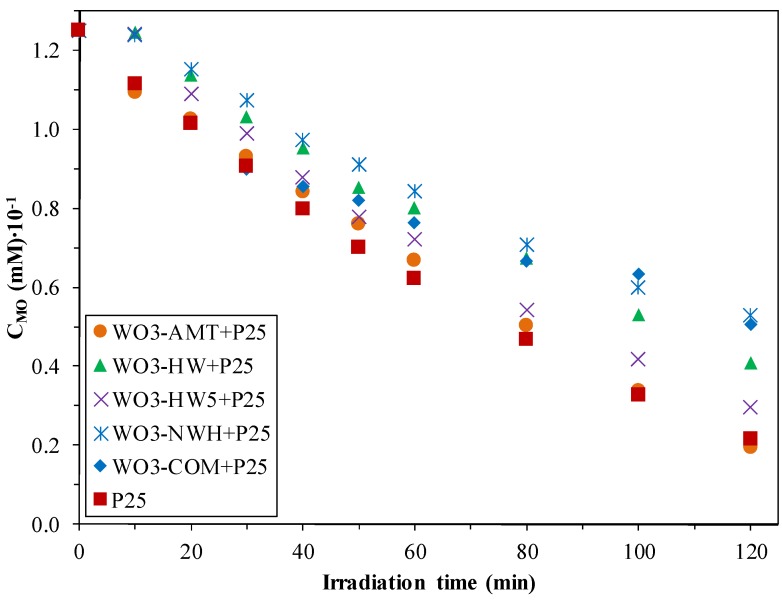
Degradation curves of methyl-orange using WO_3_-P25 composites under UV light, after 2 h.

From Figure 4, it was observed that the WO_3_-P25 composites showed different photocatalytic activities. The most important aspect was that, in the first hour of the photodegradation tests, the MO concentration decreased linearly. After 2 h, WO_3_-NWH + P25 degraded 57.7%, WO_3_-COM + P25 – 59.5%, WO_3_-HW + P25 – 67.3% of the total MO. The two best performing nanocomposites were WO_3_-HW5 + P25 (76.3% degraded MO) and WO_3_-AMT + P25 (84.6% degraded MO), while P25 removed 82.8% of the MO (Table 1).

The highest reaction rate was shown by WO_3_-COM + P25 (5.02 mmol·dm^−3^·min^−1^) and the lowest by WO_3_-NWH + P25 (0.35 mmol·dm^−3^·min^−1^). Although there was a significant difference between the two reaction rates, they only degraded ≈ 60% of MO, emphasizing again the importance of the degradation pathway of a given model pollutant. Similar incoherence was observed when comparing WO_3_-AMT + P25 and P25 (84.6% *vs.* 82.8% MO degradation/ 1.66 mmol·dm^−3^·min^−1^, 2.26 mol·dm^−3^·min^−1^). However, there are cases, when the obtained reaction rates and degradation yields showed a similar trend: WO_3_-HW + P25 (1.01 mmol·dm^−3^·min^−1^) *vs.* WO_3_-HW5 + P25 (1.06 mol·dm^−3^·min^−1^) and 67.3% MO degradation *vs.* 76.3% MO degradation.

As it was shown in this section, based upon the photodegradation results, the following main question arises: If the base photocatalyst was the same in all of the cases (Evonik Aeroxide P25) and the composites’ composition was also constant, which morpho-structural parameter was responsible for the different photocatalytic activity?

## 3. Discussions of the Photocatalytic Activity Results in the Frame of the Structural and Morphological Features

### 3.1. Morphological Aspects of the Obtained WO_3_ Microcrystals

**Figure 5:**
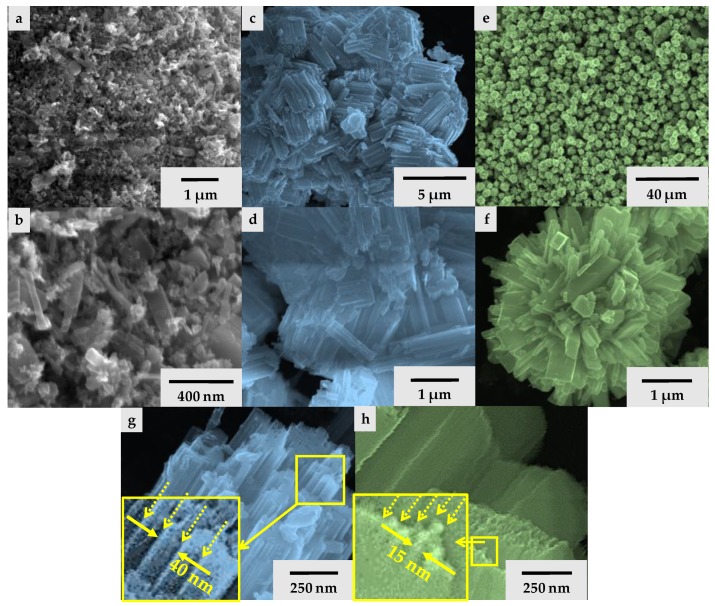
SEM micrographs of WO_3_-HW, WO_3_-HW5 (**a**;**b**); WO_3_-NWH (**c**;**d**;**g**); and WO_3_-AMT (**e**;**f**;**h**)—the yellow dotted arrows are marking the wire boundaries.

The morphology of the WO_3_ (WO_3_-HW; WO_3_-HW5) crystals synthesized from tungstic acid was rod-like, accompanied sometimes by nanosheets (Figure 5). The crystal size was ≈1 μm, which were built from very small polycrystalline nanoparticles with *d* ≈ 20 nm. This material “construction” was also observed by Liang Zhou and coworkers [20]. Using sodium tungstate as the precursor, the morphology of the tungsten trioxide (WO_3_-NWH) crystals were fiber-like [21]. Their individual length was ≈3–4 μm. Taking a closer look, it was observed that these fibers were, in fact, fiber bundles (“built” from ≈12–14 smaller nanofibers) composed from much smaller *d* = 40–50 nm fibers. Finally, the morphology of the microcrystals (WO_3_-AMT) obtained from ammonium metatungstate (AMT) was star-like [1]. These stars’ mean diameter was ≈3–4 μm and were composed from microfibers of ≈3–4 μm length. These were built from several smaller nanowires with a diameter =10–15 nm (Figure 5).

### 3.2. Crystalline Structure of the Shape-Tailored WO_3_

**Figure 6:**
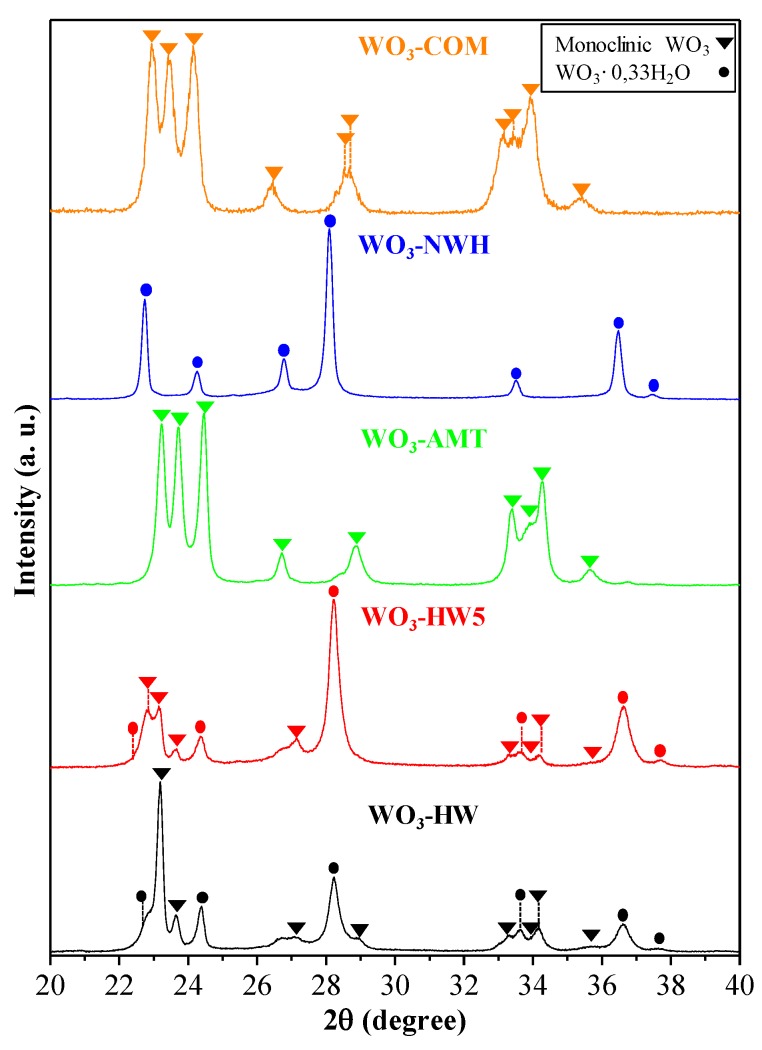
XRD patterns of the obtained WO_3_.

From the XRD patterns, the crystal phase composition and crystal size of the WO_3_ nanocrystals were evaluated. WO_3_-COM and WO_3_-AMT contained only the monoclinic crystal phase, while WO_3_-NWH contained exclusively WO_3_·0.33H_2_O hexagonal partial hydrate. Interestingly, WO_3_-HW and WO_3_-HW5 semiconductors contained both of the previously mentioned crystal phases in different amounts (Figure 6, Table 1). The crystal size values determined from the XRD patterns were well-correlated with the observations made in the previous section of the paper (except for the WO_3_-COM, which was not shown separately; the determined crystal size was 20 nm). More precisely, in the case of the hierarchically structured materials (stars made from thin wires, WO_3_-AMT and wire bundles made from smaller wires, WO_3_-NWH), the small fibers’ diameter values determined by XRD (55 nm, WO_3_-NWH; 30–35 nm, WO_3_-AMT) were in the same range as the ones determined by SEM (40–50 nm, WO_3_-NWH; 10–15 nm, WO_3_-AMT). The differences in the values can be attributed to the fibers’ asymmetrical nature (length/diameter ratio is extremely high—10–15 nm *vs.* 2–3 µm in sample WO_3_-AMT). Another important aspect was noticed when WO_3_-HW and WO_3_-HW5 was compared. If the H_2_O_2_ amount was high (WO_3_-HW), the monoclinic phase was present in 9.6 wt.%, while the hexagonal hydrate was 90.3 wt.%. When the H_2_O_2_ content was lowered, the hexagonal hydrate was still the dominant crystal phase of the powders with a more pronounced content of monoclinic WO_3_: 63.6 wt.% *vs.* 36.3 wt.% (monoclinic WO_3_).

It is known that the monoclinic crystal phase of WO_3_ is very stable, and it is thermodynamically favored if no chemical “constraints” (e.g., shaping agents) are present during the crystallization procedure. If a partial hydrate, such as WO_3_·0.33H_2_O, is desired, then a high ionic strength medium is required, where the ionic strength is determined by a joint cation and foreign anion (e.g., Na^+^/Cl^−^ Na_2_WO_4_—precursor/NaCl ionic strength modifier). These strategies were proven to be efficient, as it was shown in Figure 6 and Table 1. However, to modify the ratio of these two crystal phases, a more elaborate method is required, such as the intermediate peroxo-complex approach, which yields a different ratio of the two crystal phases depending on the H_2_O_2_ content, and it was also proven to be successful. Therefore, the next step is to verify if this crystal phase/morphology changes are related to the materials’ optical properties (band-gap value).

### 3.3. Optical Properties of the Individual WO_3_ and Composites

**Figure 7:**
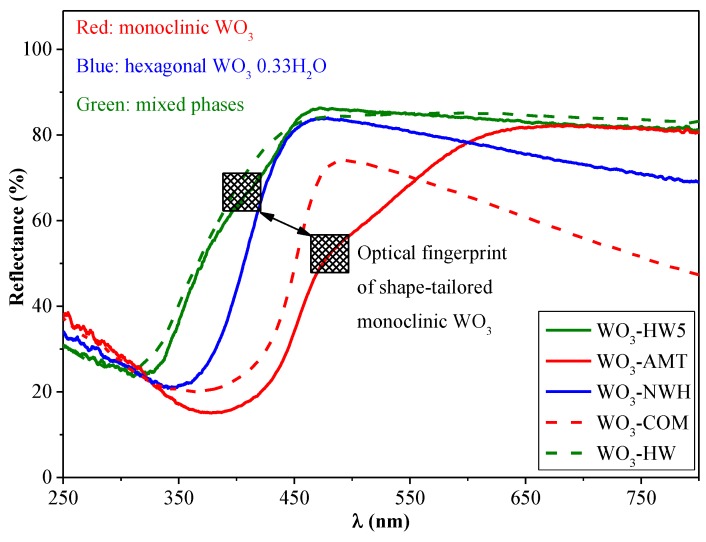
The reflectance spectra of the commercial and synthesized WO_3_.

**Figure 8:**
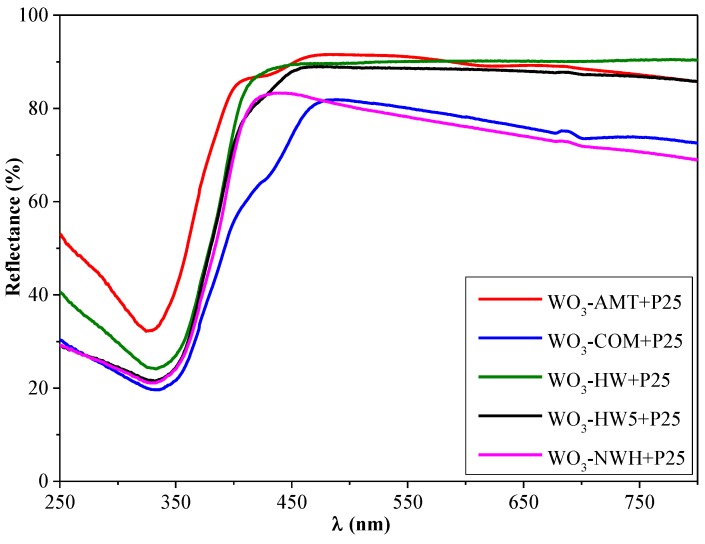
The reflectance spectra of the commercial and synthesized WO_3_-TiO_2_ composites.

The band-gap value estimated using the light absorption threshold was 450 nm (2.75 eV) for WO_3_-HW5, 460 nm (2.69 eV) for WO_3_-NWH, 475 nm (2.61 eV) for WO_3_-COM, and 550 nm (2.25 eV) for WO_3_-AMT. The lowest band-gap energy was estimated for WO_3_-AMT (interestingly, there was a break in the light absorption threshold of this material, which may require additional experimental work to be explained) and WO_3_-COM, both of them containing only the monoclinic polymorph of WO_3_. This was followed by the pure hexagonal partial hydrate containing WO_3_-NWH, and, finally by the WO_3_-HW and HW5, which contained both of the previously mentioned crystal phases. Figure 7 shows that, as the two phases were simultaneously present, a unique synergistic change was observable in the UV-Vis spectra, marked by an intensive blue shift of 50 nm (compared to WO_3_-NWH) and 100 nm (compared to WO_3_-AMT). Furthermore, in WO_3_-HW5, a more significant amount (36.3 wt. %) of monoclinic WO_3_ was also evidenced, and it was marked in the spectrum by a small break in the absorption threshold (visible also in the spectrum of WO_3_-AMT). In the case of the WO_3_-P25 composites, the band-gap values established were further blue shifted, due to the presence of TiO_2_, although the spectral features of the WO_3_ were still discernible (Figure 8). The band-gap values were summarized in Table 1.

### 3.4. The Structure-Morphology-Photocatalytic Activity Relationship

The correlation between the observed photocatalytic activities and the investigated parameters can be made at three different levels, each of them suggesting new investigation pathways concerning WO_3_ containing nanocomposites activity-tuning possibilities.

The first approach, which was already discussed in Section 2.1, was the visible light activity potential and the light absorption properties’ relationship (Section 3.3). Although all the bare WO_3_ showed visible light absorption properties (including the fact that their band-gap values were in the visible light region), no visible light activity was observable, neither in the degradation of phenol nor in the degradation of oxalic acid. Additionally, the composites prepared with P25 were also totally inactive under visible light. This result points out the fact that the WO_3_ crystals main role was in the charge separation process.

**Figure 9:**
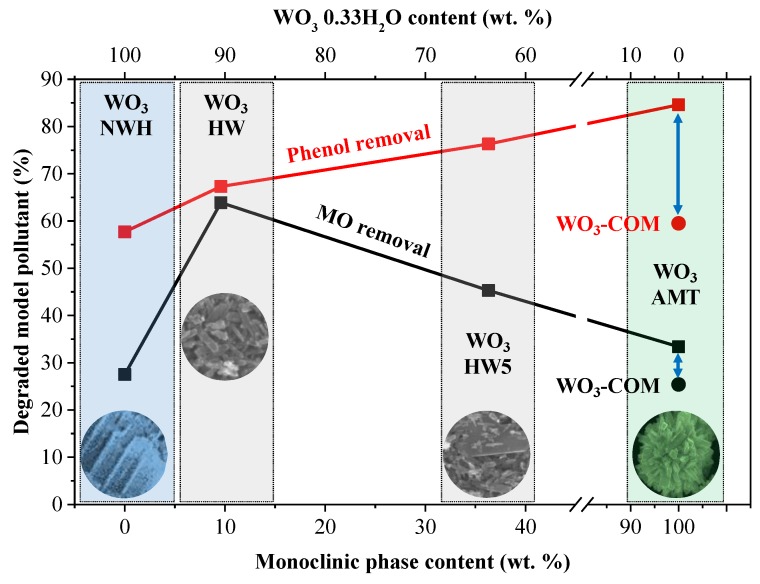
Degradation efficiencies *vs.* crystal phase composition/morphology.

The second level approach considers the relationship between the crystals’ structure and the obtained photocatalytic efficiencies. It is already known in the case of TiO_2_ that the photocatalytic activity is strongly dependent on the crystal phase composition (the famous anatase/rutile ratio— perfect synergism of the two crystal phases in P25). Therefore, a similar behavior was expected, if the crystal phase composition of the charge separator composite component (in the present case, WO_3_) was altered. In the case of phenol degradation, a small amount (≈9 wt. %) of monoclinic WO_3_ was sufficient to boost (doubling the efficiency) the activity of WO_3_·0.33H_2_O. If the amount of the monoclinic crystal phase increased further, the activity decreased gradually (Figure 9). The crystal phase composition had a reverse effect when the chosen model pollutant was MO. Pure monoclinic WO_3_ was the best choice to achieve maximum efficiency, because, with the increase of the WO_3_·0.33H_2_O content, the activity decreased gradually.

The third level approach lies in the morphological control of the WO_3_ crystals. The most representative evidence for the efficiency of shape tailoring was shown in Figure 9. WO_3_-COM + P25 showed lower photocatalytic efficiency in every one of the investigated cases (phenol and MO degradation). Both WO_3_-COM and WO_3_-AMT contained only monoclinic WO_3_, and their crystal size was in the same range. The main difference was in the fact that WO_3_-AMT contained uniform microstars that were formed from very fine nanowire bundles. This hierarchical build-up makes possible a high efficiency charge transport, which favors the separation of the photogenerated charge carriers. Furthermore, this property is exploitable not just in photocatalysis but also in development of gas sensors.

## 4. Materials and Methods

### 4.1. Chemicals

Tungstic acid (H_2_WO_4_, Sigma Aldrich, 99%), sodium tungstate dihydrate (Na_2_WO_4_·2H_2_O, Sigma Aldrich, 99%), ammonium metatungstate hydrate (AMT) ((NH_4_)_6_H_2_W_12_O_40_·xH_2_O, Sigma Aldrich, 99.99%), hydrogen peroxide (H_2_O_2_, Sigma Aldrich, 30%), hydrochloric acid (HCl, NORDCHIM, 37%, 12 M), sodium chloride (NaCl, NORDCHIM, 99.5%) were used as received. For the determination of the photocatalytic activity aqueous solution of phenol (C_6_H_5_OH, 99%, Reanal), and oxalic acid (C_2_H_2_O_4_, Aldrich, 98%) was used.

### 4.2. Synthesis of the WO_3_ Semiconductors

#### 4.2.1. Synthesis of WO_3_ Nanoplates-Intermediate Peroxo-Complex Approach

For the experiment, 2.5 g of H_2_WO_4_ was dissolved in a mixture of 30 mL 30 wt. % hydrogen peroxide (H_2_O_2_) and 10 mL of distilled water under stirring (24 h) to form a clear, pale-yellow solution [20]. 2.5 g of H_2_WO_4_ was dissolved in a mixture of 20 mL 30 wt. % hydrogen peroxide (H_2_O_2_) and 20 mL of distilled water under stirring (24 h) to form a clear/ colorless solution. Then, both of the solutions were hydrothermally treated at 180 °C for 24 h, and a white colloidal suspension was obtained. The products were collected and washed by centrifugation for 3 × 10 min at 5000 rpm, with distilled water. The washed precipitate was dried at 40 °C for 24 h. The nanocrystallites synthesized from tungstic acid were named WO_3_-HW and WO_3_-HW5. The HW abbreviation comes from the tungstic acid’s molecular formula H_2_WO_4_.

#### 4.2.2. Synthesis of WO_3_-High Ionic Strength Approach

For this part of the experiment, 3.29 g of Na_2_WO_4_·2H_2_O and 1.16 g of NaCl were dissolved in 75 mL distilled water under stirring. The pH of the suspension was adjusted to 2 with 3 M HCl aqueous solution. The suspension was stirred at room temperature for 24 h. Then, the mixture was hydrothermally treated at 180 °C for 24 h, and a green precipitate was finally obtained. The obtained product was collected and washed by centrifugation: for 3 × 10 min at 5000 rpm with distilled water. After centrifugation, the product was dried at 40 °C for 24 h [21]. The sample obtained from sodium tungstate dihydrate was named WO_3_-NWH. The NWH abbreviation comes from the sodium tungstate dihydrate molecular formula Na_2_WO_4_·2H_2_O.

#### 4.2.3. Synthesis of WO_3_ Nanostars-Low Mobility Anion Approach

For this part of the experiment, 0.77 g AMT and 0.53 mL HCl was dissolved in 12.5 mL of distilled water. The solution was stirred for 15 min, then hydrothermally treated at 180 °C for 4 h, and a yellow colloidal suspension was obtained. The product was collected and washed by centrifugation at 1600 rpm for 15 min with distilled water. After centrifugation, the precipitate was dried for 6 h at 70 °C. Finally, the as-obtained powders were thermally treated at 500 °C for 30 min [1]. The catalyst obtained using ammonium metatungstate hydrate was named WO_3_-AMT. The AMT abbreviation originates from the ammonium metatungstate hydrate.

### 4.3. Synthesis of the WO_3_/TiO_2_ Nanocomposites

In this case, the shape controlled WO_3_ nanocrystallites and Evonik Aeroxide P25 (Manufacturer, City, Country) were used for the preparation of the nanocomposites. In each case, a specific ratio was established between the composite components, according to our recent work: 24% WO_3_ and 76% TiO_2_ (Evonik Aeroxide P25). The nanocomposites were prepared via mechanical mixing in an agate mortar for 3 × 5 min [6] and were named as follows: WO_3_ name + P25. The Evonik Aeroxide TiO_2_ will be referred to as P25 later on, while the commercial WO_3_ will be denoted as WO_3_-COM. The commercial tungsten trioxide was used as a reference due to its property that it was not synthesized via hydrothermal treatment, and it doesn’t contain shape tailored WO_3_ nano and microcrystals.

### 4.4. Methods and Instrumentation

#### Characterization Methods

The XRD patterns were recorded on a Shimadzu 6000 diffractometer (Shimadzu Corporation, Kyoto, Japan), using Cu-Kα irradiation, (*λ* = 1.5406 Å), equipped with a graphite monochromator. The crystal phase of the tungsten trioxide was evaluated and the crystallites’ average size was calculated using the Scherer equation [36].

For measuring the DRS spectra of the samples, a JASCO-V650 spectrophotometer (Jasco Inc., Easton, MD, USA) with an integration sphere (ILV-724) (Jasco Inc., Easton, MD, USA) was used (*λ* = 250–800 nm). The band gap was determined according to references [34,35,37,38].

SEM micrographs were obtained with a FEI Quanta 3D FEG Scanning Electron Microscope (FEI Inc., Dawson Creek, Canada), operating at an accelerating voltage of 25 kV.

The photocatalytic tests were performed under UV irradiation in a photoreactor (homemade) (1 g·L^−1^ suspension concentration, continuous air flow, continuous stirring. 6 W × 6 W UV fluorescent lamps, *λ*_max_ = 365 nm, thermostated at 25 °C) under visible irradiation in a photoreactor (1 g·L^−1^ suspension concentration, continuous air flow, continuous stirring. 4 W × 24 W fluorescent lamps, *λ* > 400 nm, thermostated at 25 °C) [18]. The suspension containing the photocatalyst and the pollutant (initial concentration of phenol *C*_0, phenol_ = 0.5 mM or oxalic acid *C*_0, oxalic acid_ = 5 mM of methyl orange (MO) *C*_0, MO_ = 125 μM; catalyst concentration *C*_photocatalyst_ = 1 g·L^−1^; total volume of the suspension *V*_susp_ = 100 mL) was continuously purged with air, assuring a constant dissolved oxygen concentration during the whole experiment. The chosen compounds are stable under UV-A, not showing any sign of photolytic (in the absence of the photocatalyst) degradation [37].

Prior to the degradation experiments, the used suspension was kept in the dark for 10 min to establish the adsorption/desorption equilibrium. For the calculation of the reaction rates, only the first five measurement points (where the influence of the degradation intermediates was insignificant) were considered, applying a pseudo-first order kinetic approach. The error of the photocatalytic degradation experiments was verified by 3 degradation experiments with the same catalyst. The maximum error (in the conversion and reaction rate values) was determined to be ±2.5%.

The concentration decrease of the chosen organic substrate (oxalic acid, phenol) was followed using an Agilent 1100 series HPLC system (Agilent Technologies, Santa Clara, CA, USA). The eluent in the case of oxalic acid was a 0.06% aqueous solution of sulfuric acid, with a 0.8 mL·min^−1^ flow rate, the column was Grom Resin ZH (Dr. Maisch HPLC GmbH, Ammerbuch-Entringen, Germany). In the case of phenol, the eluent was a mixture of methanol and water in 7:13 ratio, while using a BST Nucleosyl C-18 column (4 mm × 250 mm) (Merck, Kenilworth, NJ, USA). The detection wavelengths were the following: in the case of oxalic acid 206 nm and in the case of phenol 210 nm. The concentration of MO was followed using a JASCO V-650 spectrophotometer at 513 nm (Jasco Inc., Easton, MD, USA).

## 5. Conclusions

The present work showed that the activity of a given nanocomposite (TiO_2_/WO_3_) can be tuned by adjusting the structural and morphological properties of the charge separator component (in the present case, WO_3_). The structural fine-tuning was efficient if the crystal phase composition was varied. This resulted in different levels of affinity towards different types of model pollutants (phenol and methyl orange). The controlled shape manipulation was also a viable alternative to enhance the photocatalytic activity, which was proven by the comparison of commercial WO_3_ and shape-tailored WO_3_ containing the same crystal polymorph and having a similar crystal size. Although there are still questions unanswered in the present research, it is clear that there is huge potential in the photocatalytic activity enhancement by applying the approaches investigated in this work.

## Figures and Tables

**Table 1:** The obtained materials’ photocatalytic activity and structural properties.

Sample Name	Structure WO_3_		Band-gap (eV)	*η*_phenol_ (%)	*r*_0, phenol_ (mM·min^−1^)	*η*_MO_ (%)	*r*_0, MO_ (mM·min^−1^)
	*MC	#HY					
P25	–	–	3.11	86.8	8.90 × 10^−3^	82.8	2.26
WO_3_-HW5	36.3	63.6	2.69	0	–	0	–
WO_3_-HW	9.3	90.6	2.75	0	–	0	–
WO_3_-NWH	0	100	2.69	0	–	0	–
WO_3_-AMT	100	0	2.25	0	–	0	–
WO_3_-COM	100	0	2.61	0	–	0	–
P25 + WO_3_-HW5	–	–	3.04	66.7	8.86 × 10^−3^	76.3	1.06
P25 + WO_3_-HW	–	–	3.00	87.2	6.53 × 10^−3^	67.3	1.01
P25 + WO_3_-NWH	–	–	2.97	44.4	5.31 × 10^−3^	57.7	0.35
P25 + WO_3_-AMT	–	–	3.10	58.7	6.69 × 10^−3^	84.6	1.66
P25 + WO_3_-COM	–	–	2.94	49.1	11.18 × 10^−3^	59.5	5.02

*MC—monoclinic WO_3_; #HY—WO_3_·0.33H_2_O.
